# Renal allograft function in kidney transplant recipients infected with SARS-CoV 2: An academic single center experience

**DOI:** 10.1371/journal.pone.0252979

**Published:** 2021-06-10

**Authors:** Skylar L. Nahi, Aneesha A. Shetty, Sajal D. Tanna, Joseph R. Leventhal

**Affiliations:** 1 Department of Surgery (Organ Transplantation), Northwestern University, Chicago, IL, United States of America; 2 Department of Nephrology and Hypertension, Northwestern University, Chicago, IL, United States of America; 3 Department of Infectious Diseases, Northwestern University, Chicago, IL, United States of America; University of Toledo, UNITED STATES

## Abstract

**Background:**

Kidney transplant recipients are a unique cohort in regard to SARS-CoV 2 susceptibility and clinical course, owing to their immunosuppressed state and propensity for kidney injury. The primary purpose of this study is to ascertain if, in kidney transplant recipients, SARS-CoV 2 infection impacts long term renal allograft function.

**Methods:**

This retrospective, single-center study reviewed 53 kidney transplant recipients with a positive SARS-CoV-2 PCR at NMH from January 1, 2020 to June 30, 2020.

**Results:**

Change in eGFR from baseline kidney function prior to infection to 90 days after the first positive SARS-CoV 2 test was +1.76%, -17.5% and -23.16% the mild, moderate and severe disease groups respectively. There was a significant decline in kidney function in the moderate and severe disease cohorts as compared to the mild disease cohort, with respective p values of p = 0.0002 and p = 0.021. Relative to the mild disease cohort, the moderate and severe disease cohorts also demonstrated significantly increased risk of developing AKI (66%, 85%), both with p values of P = 0.0001.

**Conclusions:**

Clinically severe SARS-CoV 2 infection is associated with greater risk of acute kidney injury and greater decline in renal allograft function at 90 days post infection, compared to mild disease.

## Introduction

As the health care system adapts to the COVID 19 pandemic, an understanding of disease progression and prognosis in specific patient cohorts will allow for targeted and personalized care. Transplant recipients are of particular interest, as the chronic use of immunosuppression impairs the ability to fight off viral infection, thus increasing their susceptibility to the SARS-CoV 2 virus [1, 2]. Kidney transplant patients commonly present with comorbidities including hypertension, diabetes mellitus, and cardiovascular disease, which have been demonstrated to be risk factors associated with worse SARS-CoV 2 severity and higher mortality [3]. This novel, viral pathogen, infecting a patient in an immunocompromised state, likely superimposed upon underlying comorbid conditions, may portend more severe disease and significant risk to the transplanted organ.

The immune compromised state of renal transplant recipients has an unclear impact on their SARS-CoV 2 viral susceptibility. In historical epidemics of SARS-CoV and Middle East Respiratory Syndrome viral disease, two microbially related diseases, immunosuppressed patients did not present worse outcomes than the general population, and immunosuppression was not identified as a risk factor for poor prognosis [4–6]. However, the Centers for Disease Control and Prevention (CDC) lists immunocompromised patients, including those requiring immunosuppression following renal transplantation, as high risk for severe disease from SARS-CoV-2 [7]. Indeed, while steroids have been shown to benefit some populations of patients, recent studies have indicated the routine use of corticosteroids to treat patients with SARS‐CoV‐2 may not be recommended [7]. Therefore, immunosuppressive regimens remain a challenge for patients and physicians in the Covid era [8]. More data is needed to assess how to best balance protecting a transplanted organ from a native immune system, while allowing the native immune system to protect itself from a new viral challenge.

The kidney is an organ of particular interest when considering damage associated with SARS CoV- 2 infection. Acute kidney injury (AKI) has been reported in up to 20–40% of critically-ill patients, and is directly associated with high mortality [9, 10]. Not only is it prevalent, but AKI has subsequently been associated with worse prognosis [10]. Proposed pathophysiological mechanisms include hypovolemia, nephrotoxic drugs, high PEEP, right heart failure, a direct viral injury, an imbalanced Renin-Angiotensin-Aldosterone System (RAAS) activation, an elevation of pro-inflammatory cytokines elicited by the viral infection, and a procoagulant state [11]. However, a specific mechanism has not yet been demonstrated, and importantly, the implication and long term effect of this damage has not been characterized.

Renal transplant recipients constitute a critical patient cohort when considering this viral pathogen with a predilection for the kidney. Currently, there are limited specific data on the impact of SARS-CoV-2 infection in patients with generalized immunosuppression and transplantation [3]. For this reason, the proper management of patients with kidney transplants diagnosed with COVID-19 is still being evaluated [12]. Understanding the risks posed to specific transplanted organs will allow for organ protection protocols to be developed. Organ protection protocols may include guidance on identifying risk factors, how and when to modulate immunosuppressing therapies, and monitoring periods of higher risk with specific disease markers [9].

It has been proposed that a more severe disease course may serve as a prognostic indicator of worsening kidney function, and potentially even establishment of a new, lower, baseline kidney function after recovery from the virus. Decreasing baseline kidney performance places patients at risk of earlier allograft failure requiring re-listing for a new kidney transplant, or even, in severe cases, increased risk of mortality. As such, characterizing this patient population, and the evolution of their kidney function, will serve to help care teams better understand the needs of this patient cohort, and ideally have more data on how to best protect renal functioning.

In this report, we describe our institution’s experience with SARS-CoV 2 positive kidney transplant recipients, and assess the impact of mild, moderate, and severe SARS-CoV 2 disease on renal allograft function.

## Materials and methods

This retrospective, single-center study included kidney transplant recipients with a positive SARS-CoV-2 PCR at Northwestern Memorial Hospital from January 1, 2020 to June 30, 2020. Fifty-three patients met the inclusion criteria. Each patient’s medical record was examined for key variables to elucidate patient demographics/characteristics/potential risk factors, modulation of immunosuppression over the course of disease, presentation and prognostic variables, and laboratory measures of evolving kidney function pre-, peri-, and post- infection. The data collected was then synthesized and analyzed to identify patterns and irregularities of the cohort.

Each patient’s clinical disease severity was ranked according to the WHO Ordinal Scale for SARS-CoV-2 Clinical Improvement (WHO R&D Blueprint, novel Coronavirus, COVID-19 Therapeutic Trial Synopsis, WHO 2020). The study population was divided into three groups based on disease severity (mild disease (ordinal scale of 0–2): n = 11, moderate disease (ordinal scale of 3–4): n = 29, severe disease (ordinal scale of 5–8): n = 13). The primary endpoint was change in estimated GFR (eGFR), employing the Chronic Kidney Disease Epidemiology Collaboration (CKD-EPI) Creatinine algorithm, 90 days after the positive SARS-CoV 2 PCR. Secondary endpoints for this study included observation for development of AKI, observation for proteinuria, ability to demonstrate two negative tests within 90 days of infection, and possible rehospitalization. Relevant demographic and clinical data was also collected. The investigators used a chi-squared analysis for categorical variables, and a Welch’s T-test to assess differences between sets of two cohorts. A one-way ANOVA test was employed to compare the 3 groups.

The authors secured Institutional review board (IRB) approval and confidentiality was ensured with depersonalized data and encrypted files. Once identified in Epic (EHR), patient data was transferred to a deidentified, password-protected spreadsheet that was encrypted and saved to a password protected drive. The data collection process concluded in September 2020. The data was accessed and processed from this twice encrypted site, and never transferred elsewhere. The investigators received a waiver of consent, as the research involved no more than minimal risk to participants, would not affect participants’ rights or welfare, and the research could not practically be carried out without the waiver. Study approved by the Northwestern University Institutional Review Board Office: IRB approval number: STU00213729.

## Results

### Patient characteristics

In the total patient cohort, prevalence of baseline characteristics/risk factors is as follows: HTN (100%), diabetes (55%), obesity (BMI ≥ 30.0) (42%), advanced age (≥60 years) (34%), and heart disease (26%). The severe clinical disease cohort presented with statistically elevated rates of advanced age and comorbid diabetes mellitus when compared to the mild disease cohort (P< 0.03, P<0.04) and the moderate disease cohort (p<0.04, P<0.02) (Table 1).

**Table 1:** Characteristics of kidney transplant recipients with SARS-CoV-2 at presentation.

Patient Characteristic	Mild Disease Cohort	Moderate Disease Cohort	Severe Disease Cohort	P value (a: mild vs. moderate; b: moderate vs. severe; c: mild vs. severe)
Advanced Age (> = 60)	2 (18%)	8 (28%)	8 (62%)	*0*.*04*^*b*^, *0*.*03*^*c*^
HTN	11 (100%)	29 (100%)	13 (100%)	
Diabetes Mellitus	5 (45%)	13 (45%)	11 (85%)	*0*.*02*^*b*^, *0*.*04*^*c*^
Heart Disease	2 (18%)	6 (21%)	6 (46%)	
Obesity	2 (18%)	14 (48%)	6 (46%)	
Enhanced immunosuppression	1 (9%)	6 (21%)	1 (8%)	
Proteinuria	2 (18%)	13 (45%)	7 (58%)	*0*.*05*^*c*^

(insignificant p values not reported).

**Fig 1:**
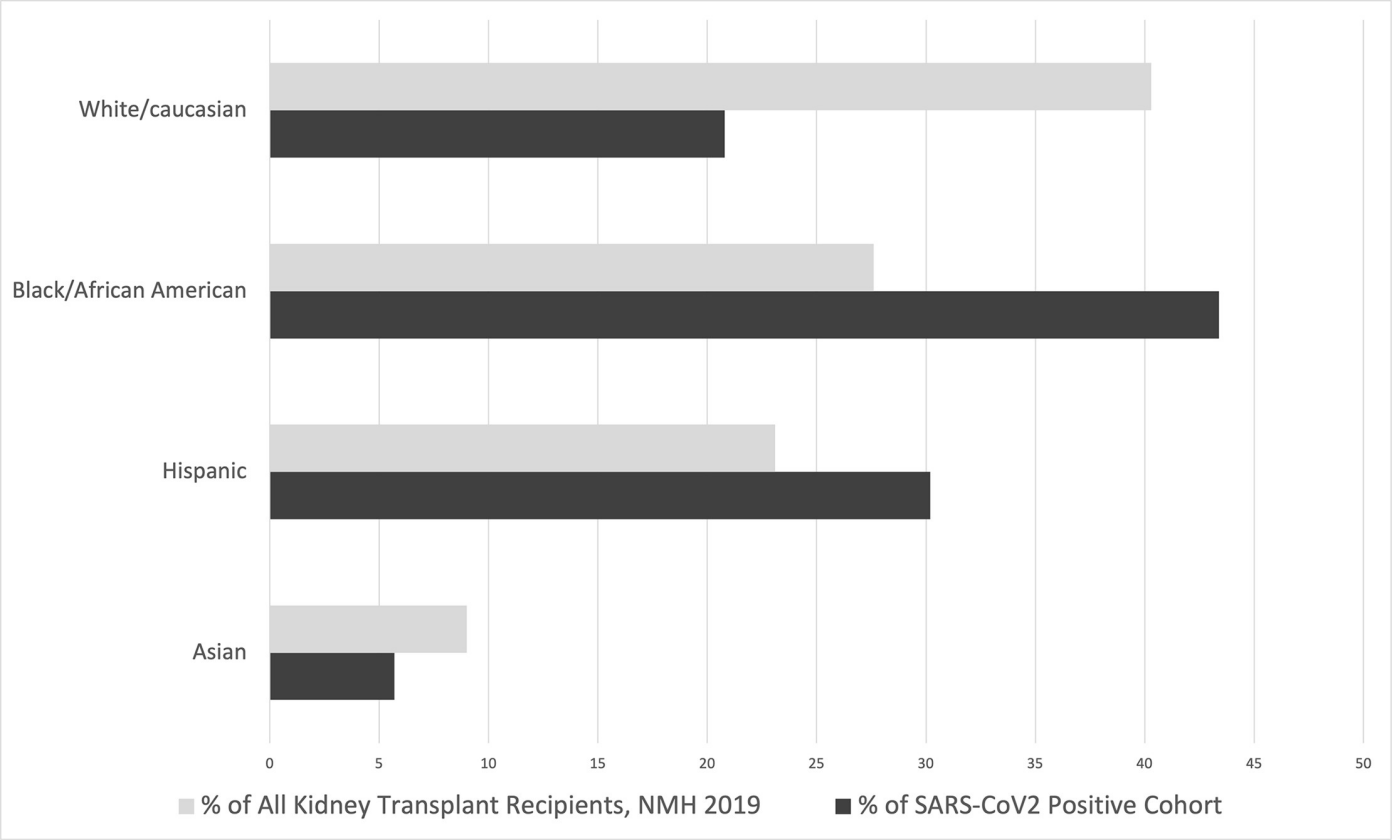
Racial discrepancies between NMH 2019 kidney transplant recipients and the SARS-CoV 2 positive kidney transplant recipient cohort.

Black American patients were disproportionately overrepresented in the SARS-CoV 2 positive cohort, as compared to rates of transplantation at NMH in 2019 (p = 0.025) (Fig 1). Hispanic patients were also overrepresented, although to a lesser degree.

### Clinical presentation

Over the course of clinical disease, proteinuria was identified in 42% of patients, significantly higher in the severe disease cohort (58% positive for proteinuria) as compared to the mild disease cohort (18% positive for proteinuria) (p = 0.05) (Table 1).

A fraction of the patients were rehospitalized within the 90 day timeframe from SARS- CoV 2 associated sequela. Of the mild disease cohort, 9%, of the moderate disease cohort 28%, and of the severe disease cohort, 46% were either rehospitalized or deceased at the 90 day end point.

Fatality rate of this total patient cohort at 90 days post infection was 7.5%. All the patients who expired were in the severe patient cohort, and thus the fatality rate in the severe patient cohort was 31%.

### Immunosuppression

Enhanced immunosuppression, defined by transplant or rejection episode treated with elevated immunosuppression within the last year, was only present in 15% of total cases, and not correlated with disease severity (Table 1).

All kidney transplant patients who were diagnosed with symptomatic COVID-19 underwent immunosuppression reduction without complete withdrawal, based on a protocol mutually agreed upon by the transplant team at our institution. Hospitalized patients underwent reduction in the antimetabolite (Mycophenolate or Azathioprine dose) while largely maintaining the Calcineurin dose. If the patient developed hypoxia and needing significant oxygen supplementation, the antimetabolite was discontinued completely. In more severe cases requiring intensive care, Calcineurin dose was reduced in addition to discontinuation of the antimetabolite. In general, Belatacept and mTOR inhibitors were avoided.

In addition to immunosuppression reduction, additional therapeutic approaches were implemented in patients determined to be eligible on a case-by-case basis. Qualitatively, empiric coverage for community acquired pneumonia was often initiated in the emergency department, and then subsequently continued, modified, or discontinued by the care team. In this patient cohort, 17 of the 53 patients received SARS-CoV 2 directed therapy, including Remdesivir, Sarilumab, Hydroxychloroquine, Tocilizumab, and convalescent plasma. In this study, the patient population treated with SARS-CoV 2 directed therapy did not yield statistically different outcomes as compared to those patients who did not receive SARS-CoV 2 directed therapy.

### Renal allograft function

**Fig 2:**
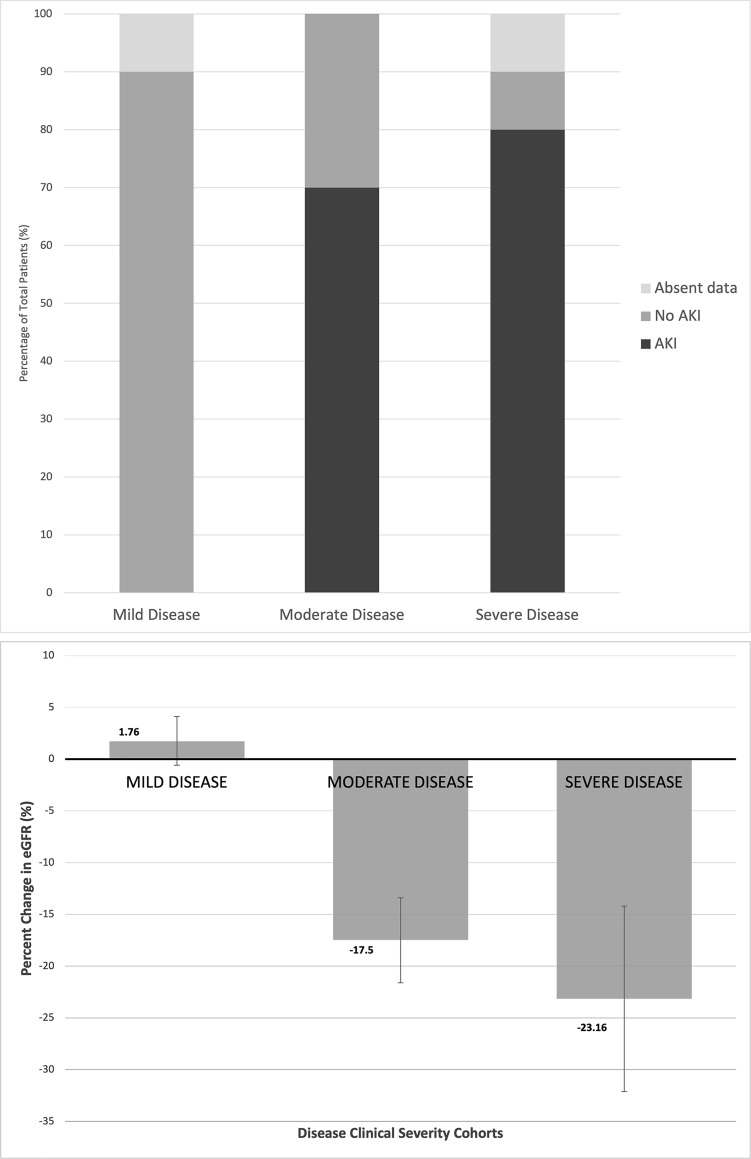
Impact of increasing clinical SARS-CoV-2 disease severity on renal allograft function. (a) Incidence of AKI developed over course of clinical infection in each of the three patient cohorts, represented as a percentage of the cohort total. (b) In each clinical disease severity cohort, percent change in estimated GFR (eGFR) from baseline kidney function prior to the positive SARS-CoV 2 PCR to 90 days after the positive test.

Change in eGFR from baseline kidney function prior to infection to 90 days after the first positive SARS-CoV 2 test was +1.76%, -17.5% (p = 0.0002) and -23.16% (p = 0.021) in the mild, moderate and severe disease groups, respectively (Fig 2). There was a significant decline in kidney function in the moderate and severe disease cohorts as compared to the mild disease cohort. Relative to the mild disease cohort, the moderate and severe disease cohorts presented with increased risk of developing AKI. In the moderate disease cohort 66% of patients developed AKI, and in the severe disease cohort 85% of patients developed AKI, both demonstrating statistically increased risk relative to the mild disease cohort with p values of P = 0.0001 (Fig 2).

To characterize the declining renal function, kidney biopsies were performed in 4 patients (of the 53 patient cohort). In two of the four, reports indicated moderate cellular rejection. One of these two patients received modification of their immunosuppression via decreasing tacrolimus dose and increasing steroid dosing. The other patient with moderate cellular rejection received a modified immunosuppression regimen decreasing dose of tacrolimus and temporarily discontinuing azathioprine. The two remaining biopsies found collapsing focal segmental glomerulosclerosis and mild borderline cellular infiltrates with mild podocytopathy, respectively.

### SARS-CoV-2 shedding

**Fig 3:**
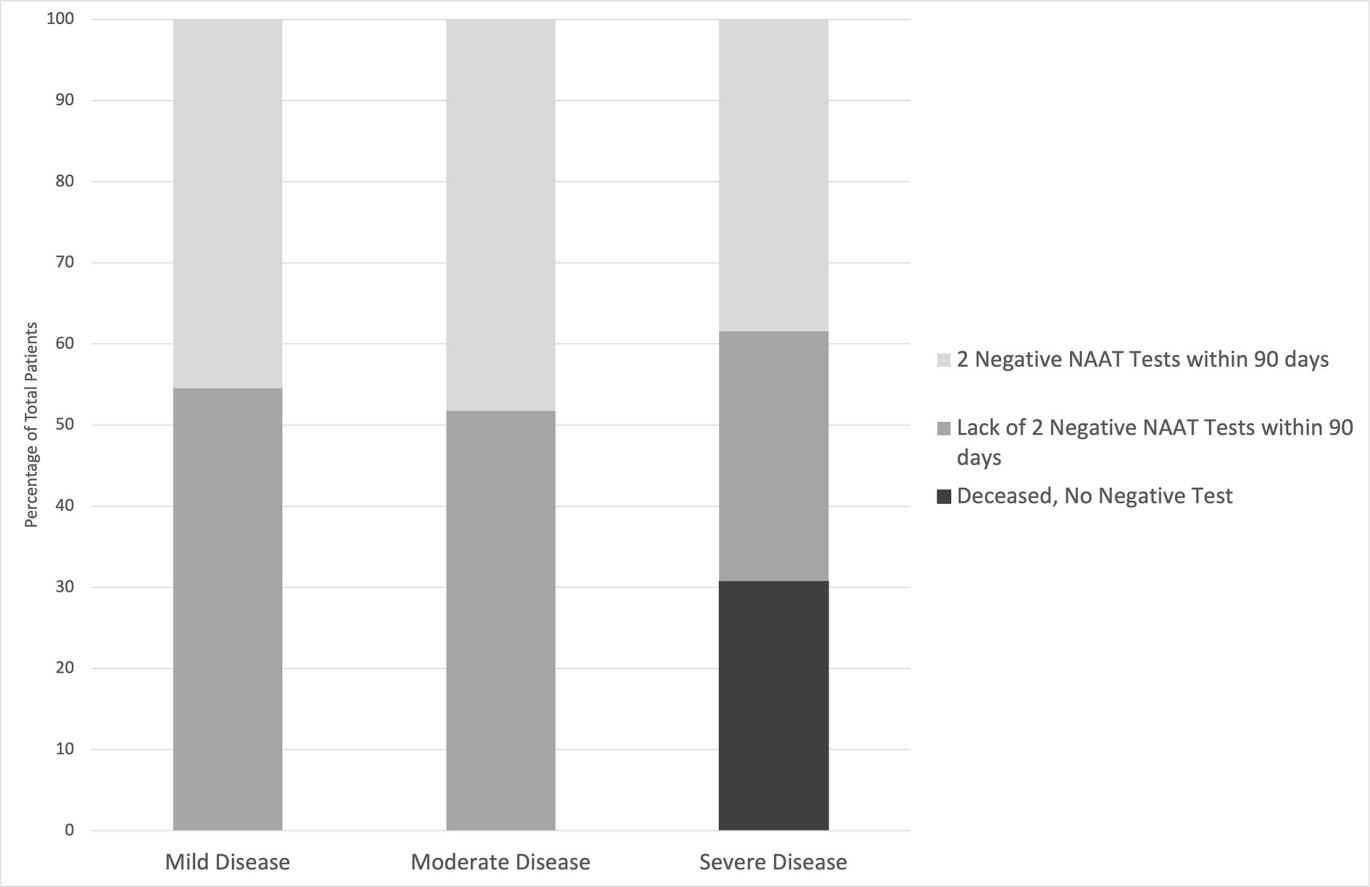
SARS-CoV2 negativity within 90 days of positive test of SARS-CoV2 infected kidney transplant recipient cohort organized by severity of clinical disease.

At the end point of 90 days after first positive test, there was no significant difference in the ability to demonstrate cessation of shedding (defined as two negative tests at least 24 hours apart). Of the mild disease cohort, 45% demonstrated cessation of shedding (n = 11); of the moderate disease cohort, 48% demonstrated cessation of shedding (n = 29), and of the severe disease cohort, 38% demonstrated cessation of shedding (n = 13) (Fig 3).

## Discussion

Kidney transplant recipients constitute a patient cohort of particular interest concerning SARS-CoV 2 infection. These patients are characterized by long-term immunosuppression, comorbidity and residual chronic kidney disease, and investigation is beginning to demonstrate increased mortality when infected with SARS-CoV 2 [13]. Infection itself puts the scarce resource of the transplanted organ at risk of AKI and organ failure, necessitating investigation into three key questions: First, of kidney transplant recipients, who is most at risk? Second, in these patients how does the course of clinical disease present, and do there exist cohort-specific prognostic indicators? Third, what is the impact of SARS-CoV 2 infection on the renal allograft functioning of these patients? This study presents a single, academic center experience, with a cohort of fifty-three patients, to address these key issues.

In consideration of groups at particular risk, this study reports disparity between the representation of racial minorities in the overall transplant recipient group at this institution, and their representation in the SARS-CoV-2 infected transplant recipient group at this institution (Fig 1). This finding is consistent with the reported pattern of overall of higher representation of Black Americans and Hispanic Americans in total SARS-CoV-2 cases and severity of illness [14]. Furthermore, these results may be impacted by decreased access to testing or the barrier of medical expenses. These possible confounders would most likely implicate the patients who are disadvantaged by social determinants of health, and thus the disparity may be even greater than reported. This finding highlights a racial health disparity within a specific patient cohort, potentially mediated by social determinants of health, identifying a need for further investigation, and closer monitoring of these patients.

Key comorbid conditions demonstrated to be most prevalent in SARS CoV-2 infected patients include hypertension, diabetes, cardiovascular disease, advanced age and obesity [15, 16]. This study demonstrates those risk factors do apply to the kidney transplant recipient cohort in isolation. These comorbidities are not only prevalent in the patient cohort, but there is also association with increasing disease severity. Specifically, advanced age and comorbid diabetes mellitus are significantly more prevalent in more severe clinical disease. These data serve as a tool to help identify kidney transplant recipients who may be at higher risk for infection, and even higher risk for experiencing more severe SARS CoV-2 disease.

Immunosuppression continues to be a patient characteristic of unclear significance in consideration of SARS CoV-2 susceptibility and severity of disease. As 100% of the patients in the studied cohort were on some degree of immunosuppression, enhanced immunosuppression (defined by transplant or rejection episode treated with elevated immunosuppression within the last year) was the variable employed to analyze how increased immunosuppression implicated SARS CoV-2 susceptibility. A minority of the patient cohort (only 15%), demonstrated enhanced immunosuppression, and those patients did not present with clinical course or outcome different from the patient population as a whole (Table 1).

These findings add to the literature, providing the Northwestern Memorial Hospital experience as a tool to guide how immunosuppression patients may be managed to best care for their health and wellness, while protecting them from SARS-CoV2 with an evidence based medicine approach. We have identified no evidence of enhanced immunosuppression worsening clinical outcomes. From these data, it may be hypothesized that lowering immunosuppression of kidney transplant recipients may not be a necessary or effective method of protection from the virus, while putting their transplanted organ at higher risk of rejection [17].

Studies in the literature have begun to look specifically at kidney transplant recipients with SARS CoV-2 with the aim of describing the course of their clinical disease. The present study demonstrates consistent findings across of number of variables, as well as analysis of how these findings vary with clinical severity. Utilization of the WHO Ordinal Scale for SARS CoV-2 illness severity allows for consistency in recording of individual outcome events, facilitates interpretation and potential future combination of results across studies and trials.

**Fig 4:**
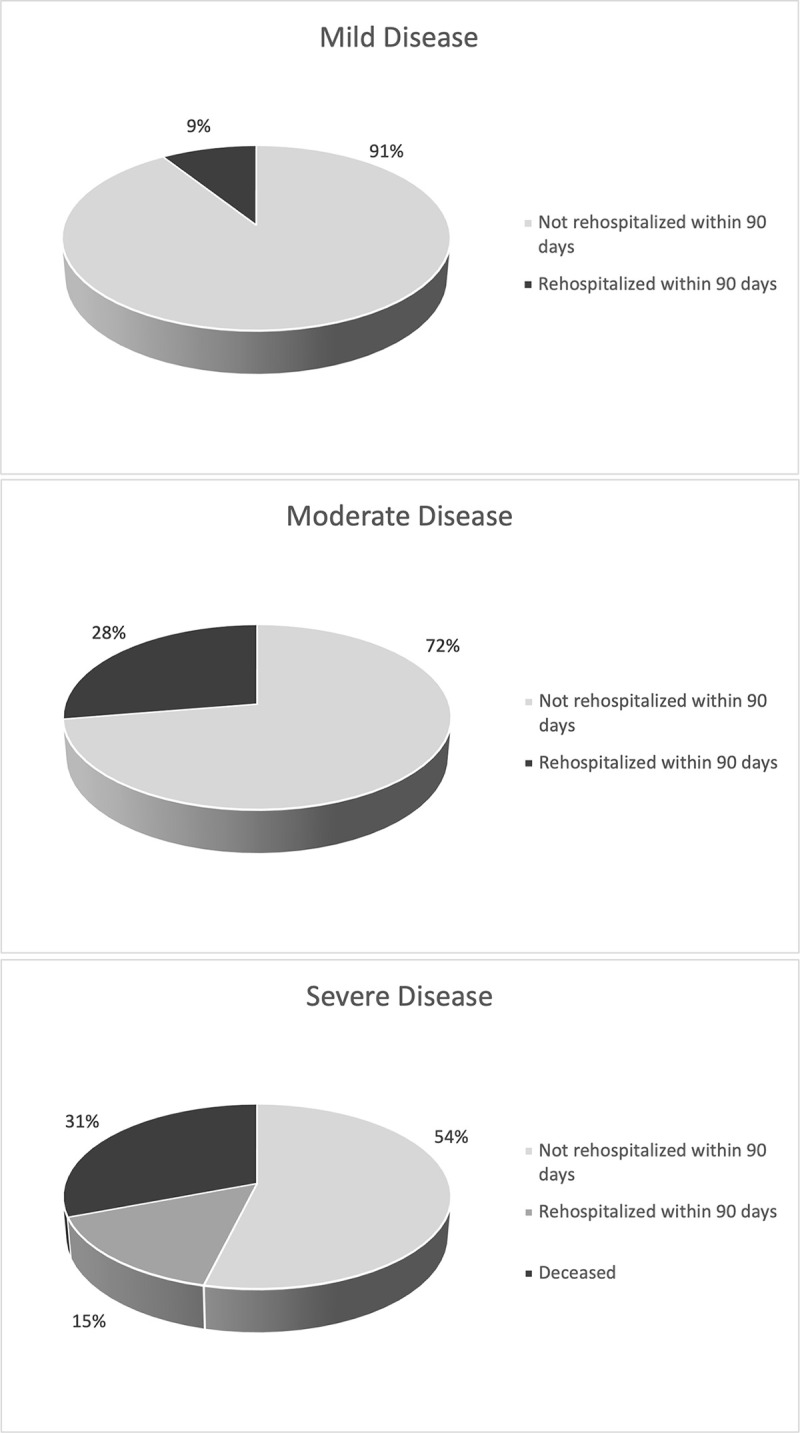
For differing severities of clinical disease, likelihood of rehospitalization for SARS-CoV sequela within 90 days of positive SARS-CoV 2 test. (a) Mild disease cohort. (b) Moderate disease cohort. (c) Severe disease cohort.

Increasing severity of clinical disease was found to be correlated with disease characteristics including likelihood of rehospitalization and death (Fig 4). Conversely, extended and variable length of the viral shedding period was not directly correlated with ordinal scale ranking. Across the severity groups, this study found varying time periods for viral shedding, with more than 50% of each disease severity subgroup unable to demonstrate cessation of viral shedding at the 90 day study end point (Fig 3). This finding, in an evidence-based manner, supports heightened isolation and quarantine guidance for the patient population. As such, further investigation on the relationship between either disease or SARS CoV-2 vaccination, on viral shedding and antibody development is warranted in the population.

It has been proposed in the literature that proteinuria may be a robust marker of SARS CoV-2 disease. This study identified a statistically significant increase in presence of proteinuria with increase in clinical disease severity (p = 0.05). Thus, it can be hypothesized that proteinuria may be a reliable prognostic indicator of disease severity in kidney transplant recipients, and even provide indication of how the kidney is acutely implicated by the viral infection.

Mortality rates for SARS CoV-2 infection continue to change and have consistently been shown to differ in specific patient subgroups. For this academic single center experience in the first six months of the SARS CoV-2 pandemic, infected kidney transplant recipients had a 7.5% fatality rate at 90 days post infection. Of consideration, this mortality figure may be confounded by undercounting of mild/asymptomatic cases, and thus be higher than a true value. This is consistent with the literature, demonstrating mortality in transplant recipients to be higher than mortality in non-transplant recipients [3, 13].

Beyond the endpoint of mortality, impact on renal allograft mortality is a vital consideration in this patient population. Consistent with the literature, this study found acute kidney injury was commonly present with SARS CoV-2 infection. Specifically, relative to the mild disease cohort, the moderate and severe disease cohorts demonstrated significantly increased risk of developing AKI, both with p values of P = 0.0001 (Fig 2). Several etiologies of this damage have been proposed, including direct cytopathic damage, immune-mediated damage, iatrogenic damage, rhabdomyolysis, impairment of renal microcirculation, and disseminated intravascular coagulation [10]. The findings of this study highlight the need to further investigate the pathophysiology of this damage.

Kidney biopsy is one approach to an investigation of the mechanism of the SARS CoV-2 virus damage to kidneys. Two of the four patients who received kidney biopsies in this patient population presented with moderate cellular rejection, which the other two presented with different patterns of damage that warrant further investigation. Members of this study’s team have identified and contributed to the literature a patient series highlighting six patients with SARS CoV-2, for whom the renal manifestation of disease was AKI, collapsing glomerulopathy and high-grade proteinuria [18]. This finding, in conjunction with the data presented in the present study, guide future investigation into the pathophysiology of disease in the kidney.

Regarding long term kidney function, this study presents novel data demonstrating moderate and severe SARS CoV-2 infection affecting kidney function 90 days after infection, bringing down baseline kidney function. Not only can the long-term function of kidney transplant recipients’ allograft be damaged and impaired, but there also exists a direct correlation between the severity of disease and the extent of the long term damage to the allograft. This novel finding adds to the current literature, supporting continued innovation in the care of this patient cohort, focusing on the preservation of their organ function.

Limitations of this study include its retrospective design, and the potential bias produced as a result of possible mild cases which were not tested and identified. In the first six months of the pandemic, testing access and knowledge of presenting symptoms likely hindered identification of all mild cases. As such, the identified population of the fifty-three kidney transplant recipients who tested positive for SARS CoV-2 at Northwestern Memorial Hospital may over-represent those with moderate to severe symptoms. This study targeted this potential bias by separating the three categories of clinical disease, and making direct comparisons between the groups.

In conclusion, this study demonstrates the association between clinically severe SARS-CoV 2 infection in kidney transplant recipients and a greater risk of acute kidney injury and greater decline in renal allograft function at 90 days post infection, compared to mild disease. These results highlight the importance of closely monitoring renal allograft function in kidney transplant patients with SARS- CoV 2 infection. Furthermore, they underscore the need for additional studies to determine longer term allograft outcomes.

## Supporting information

S1 Data Minimal data set for the study patient cohort of 53 patients at Northwestern Memorial Hospital. (XLSX)

## Abbreviations

AKI: acute kidney injury
BMI: body mass index
CDC: Centers for Disease Control and Prevention
COVID: severe acute respiratory syndrome coronavirus 2
eGFR: estimated glomerular filtration rate
HTN: hypertension
NMH: Northwestern Memorial Hospital
PCR: polymerase chain reaction
PEEP: positive end-expiratory pressure
SARS-CoV 2: severe acute respiratory syndrome coronavirus 2
WHO: World Health Organization
